# Genome Sequence of the Acidophilic Sulfate-Reducing *Peptococcaceae* Strain CEB3

**DOI:** 10.1128/genomeA.00886-15

**Published:** 2015-08-06

**Authors:** Patrick Petzsch, Anja Poehlein, D. Barrie Johnson, Rolf Daniel, Michael Schlömann, Martin Mühling

**Affiliations:** a Institute of Biological Sciences, TU Bergakademie Freiberg, Freiberg, Germany; b Genomic and Applied Microbiology & Göttingen Genomics Laboratory, Georg-August-Universität Göttingen, Göttingen, Germany; c College of Natural Sciences, Bangor University, Bangor, United Kingdom

## Abstract

We report the draft genome of the *Peptococcaceae* strain CEB3 that originated from an acidic (pH 2.5) stream draining an abandoned copper mine. Strain CEB3 is one of the very few reported acidophilic sulfate-reducing isolates. The 5.04-Mb draft genome harbors 5,069 predicted protein-encoding and 66 RNA genes.

## GENOME ANNOUNCEMENT

Acidophilic sulfate-reducing bacteria are of particular interest for the treatment of sulfate- and metal-enriched acid mine drainage (AMD) waters since sulfidogenesis is a proton-consuming process (thereby promoting amelioration of mine water acidity) and the hydrogen sulfide produced can be used to selectively precipitate transition metals present in AMD (1). Strain CEB3, which, based on its 16S rRNA gene sequence, is likely to be related to a new genus within the *Peptococcaceae* (*Firmicutes*), is a spore-forming, motile, obligately anaerobic sulﬁdogen that was isolated as one of the dominating microbial strains present in a sulfidogenic bioreactor operated at pH 2.4 (±0.1) for the selective recovery of metals (1). The inoculum for this bioreactor originated from an anaerobic streamer mat growing in an acidic (pH 2.5) stream draining an abandoned copper mine in southwest Spain (1).

Chromosomal DNA of *Peptococcaceae* strain CEB3 was isolated with the MasterPure complete DNA purification kit (Epicentre) and used to generate 454-shotgun and Illumina-shotgun libraries according to the manufacturers’ protocols. The libraries were sequenced using a 454 GS-FLX system (Titanium Chemistry, Roche Life Science) and the Genome Analyzer IIx (2×112 bp paired-end sequencing; Illumina). Sequencing resulted in coverages of 8.03 and 78.26, respectively. The assembly of the genome sequence was performed with the Roche Newbler assembler v2.9 and Mira v3.4 (2) and resulted in 64 contigs.

The draft genome of strain CEB3 comprises a single chromosome of 5.04 Mb with an overall G+C content of 52.58%. Prodigal (3) was used for automated gene prediction, and identification of rRNA and tRNA genes was carried out using RNAmmer (4) and tRNAscan (5), respectively. The IMG-ER (Integrated Microbial Genomes-Expert Review) system (6) was used for automated annotation, which was subsequently manually curated using the Swiss-Prot, TREMBL, and InterPro databases (7). The draft genome harbored 9 rRNA genes, 57 tRNA genes, and 3,898 predicted protein-encoding genes with function prediction and 1,171 genes coding for hypothetical proteins.

Analyses of the protein-coding genes revealed that strain CEB3 appears to be a metabolically versatile acidophile. For instance, strain CEB3 uses both phosphate and phosphonate (via C-P lyase activity) as a P source. Similarly, it can utilize several nitrogen sources for biomass production (nitrate, ammonium, and dinitrogen gas). The assimilated nitrogen is predicted to be stored in the form of cyanophycin, which is known to play a role in heavy-metal resistance (8).

Strain CEB3 appears to be able to utilize sulfate as a source of sulfur and as an electron acceptor for dissimilatory reduction. Utilization of trithionate and tetrathionate or thiosulfate may provide alternative avenues for sulfur acquisition and respiration, though only one subunit of the thiosulfate reductase has been detected in the genome sequence. Strain CEB3 may assimilate carbon either through CO_2_ fixation via the reductive acetyl–CoA pathway or take up organic carbon molecules via monosaccharide and disaccharide transporters.

### Nucleotide sequence accession numbers.

The results from this genome sequencing project have been deposited at GenBank under the accession number LDXJ00000000. The version described in this paper is version LDXJ00000000.1.
